# E-cadherin acts as a positive regulator of the JAK-STAT signaling pathway during *Drosophila* oogenesis

**DOI:** 10.3389/fcell.2022.886312

**Published:** 2022-08-23

**Authors:** Charlotte Mallart, Fabienne Chalvet, Sophie Netter, Alba Yurani Torres, Mickael Poidevin, Jacques Montagne, Anne-Marie Pret, Marianne Malartre

**Affiliations:** ^1^ Institute for Integrative Biology of the Cell (I2BC), CEA, CNRS, Université Paris-Saclay, Gif-sur-Yvette, France; ^2^ Institute for Integrative Biology of the Cell (I2BC), UVSQ, CEA, CNRS, Université Paris-Saclay, Gif- sur-Yvette, France

**Keywords:** JAK-STAT, signaling, oogenesis, E-cadherin, morphogenesis, apoptosis

## Abstract

The JAK-STAT pathway is evolutionary conserved. The simplicity of this signaling in *Drosophila*, due to the limited redundancy between pathway components, makes it an ideal model for investigation. In the *Drosophila* follicular epithelium, highly stereotyped functions of JAK-STAT signaling have been well characterized, but how signaling activity is regulated precisely to allow the different outcomes is not well understood. In this tissue, the ligand is secreted by the polar cells positioned at each follicle extremity, thus generating a gradient of JAK-STAT activity in adjacent cells. One way to control the delivered quantity of ligand is by regulating the number of polar cells, which is reduced by apoptosis to exactly two at each pole by mid-oogenesis. Hence, JAK-STAT activity is described as symmetrical between follicle anterior and posterior regions. Here, we show that JAK-STAT signaling activity is actually highly dynamic, resulting in asymmetry between poles by mid-oogenesis. Interestingly, we found similar temporal dynamics at follicle poles in the accumulation of the adherens junction E-cadherin protein. Remarkably, E-cadherin and JAK-STAT signaling not only display patterning overlaps but also share functions during oogenesis. In particular, we show that E-cadherin, like JAK-STAT signaling, regulates polar cell apoptosis non-cell-autonomously from follicle cells. Finally, our work reveals that E-cadherin is required for optimal JAK-STAT activity throughout oogenesis and that E-cadherin and Stat92E, the transcription factor of the pathway, form part of a physical complex in follicle cells. Taken together, our study establishes E-cadherin as a new positive regulator of JAK-STAT signaling during oogenesis.

## Introduction

**FIGURE 1:**
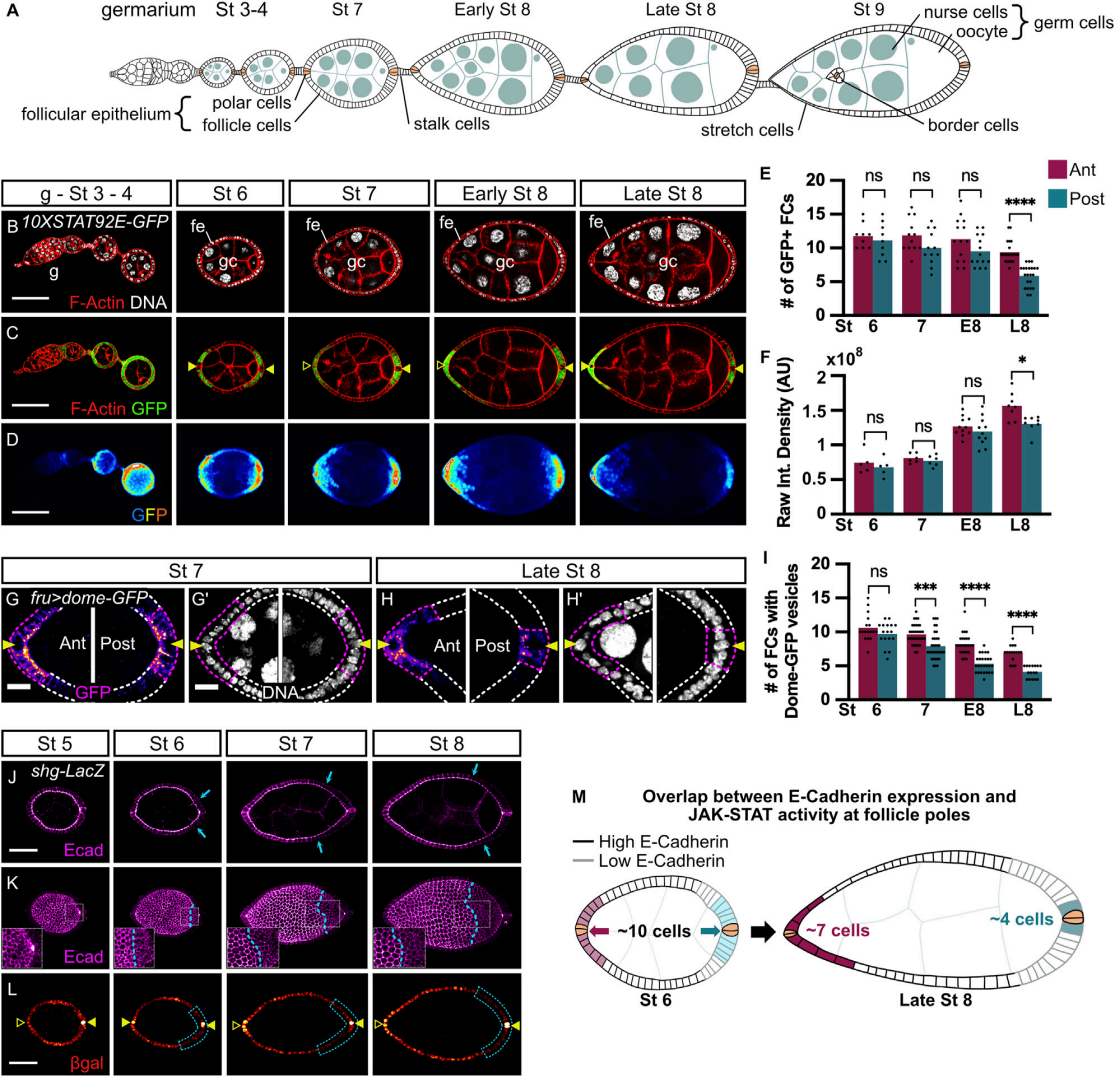
JAK-STAT signaling activity and E-cadherin expression are dynamic and asymmetric between anterior and posterior poles starting at stages 7 and 6 of oogenesis respectively. In all images and drawings, the anterior is to the left. Filled arrowheads indicate PCs. Empty arrowheads indicate the position of PCs along the *z*-axis when not visible on the displayed z section. In graphs, purple and blue bars represent mean measurements at anterior (ant) and posterior (post) poles respectively and each dot corresponds to one measurement. **(A)** Schematic drawing illustrating the organization of an ovariole with the germarium **(G)** located at the anterior-most tip and older stages towards the posterior end linked by the stalk cells. The somatic follicle monolayered epithelium (fe), composed of follicle cells (FC), including polar cells (PC, in beige), border cells, and stretch cells surrounds the cyst of 16 germ cells (gc) comprising 15 nurse cells and 1 oocyte that is anchored at the posterior pole. **(B–D)** Follicles from *10XSTAT92E-GFP* hemizygous females carrying a *STAT92E* transcriptional reporter at the indicated stages of oogenesis **(B)** F-Actin (red, phalloidin) and DNA (grey, DAPI) staining. **(C,D)** Native GFP (C: green, one confocal section; **(D)** “royal” filter, sum projection of total GFP signal) reflects JAK-STAT activity at each pole forming a gradient in FCs adjacent to PCs. **(E)** The mean number of GFP + FCs at each pole counted on one confocal section that includes PCs. From stage 7, GFP + FC number decreases at the posterior pole and is significantly lower than at the anterior pole when follicles attain late stage 8 (L8). **(F)** Raw Integrated Density quantification of 25% of follicle length starting at each pole on the sum projection of total GFP signal. At L8, GFP intensity is significantly lower at the posterior pole than at the anterior pole **(G,H)** Stage 7 and L8 follicles from females expressing *UAS-dome-GFP* in FCs with the *fru-Gal4* driver (max projection of 3–6 confocal sections of anterior and posterior follicle poles). The number of FCs active for JAK-STAT signaling (purple dotted lines) adjacent to PCs (arrowheads) was determined by counting FCs containing Dome-positive vesicles (“fire” filter, from purple, low intensity, to orange, high intensity). **(I)** Mean number of FCs containing Dome-GFP vesicles at each pole shows a progressive decrease at both anterior and posterior poles from stage 7 to L8, but more drastically at the posterior pole with significant differences with the anterior pole at all stages. **(J–L)** Follicles at the indicated stages of oogenesis from *shotgun-LacZ* (*shg-LacZ*) homozygous females carrying a *shg* transcriptional reporter. **(J)** E-cadherin immunostaining (“magenta hot” filter, from magenta, low intensity, to white, high intensity) on a max projection of 3 confocal sections shows E-cadherin apico-lateral localization within the follicular epithelium, with a weaker intensity at the posterior pole from stage 6 (blue arrows show the border between high and low E-cadherin intensity). **(K)** 3D projections of E-cadherin immunostaining (“magenta hot” filter) of the same follicles as in **(J)** highlight the alveolus-like organization of E-cadherin in the follicular epithelium. E-cadherin protein is homogenously distributed along the anterior-posterior axis up to stage 5 of oogenesis and then decreases at the posterior pole compared to the anterior pole from stage 6 (blue dotted lines indicate the border between high and low E-cadherin intensity). Insets on the posterior follicle poles are magnifications of the white dotted area. **(L)** Beta-galactosidase (βgal) immunostaining (“red hot” filter, from red, low intensity, to yellow, high intensity) on the same projection as in **(J)** shows FCs that express the *shg-LacZ* reporter reflecting the *shg* expression pattern at the transcriptional level. Like the E-cadherin protein, *shg* transcriptional activity between poles changes from homogeneous at stage 5 to lower at the posterior pole than at the anterior pole from stages 6–8 (blue dotted lines) in 100% of observed cases (*n* = 70). **(M)** Schematic drawing of stage 6 and late stage 8 follicles representing JAK-STAT signaling dynamics together with E-cadherin expression pattern in the follicular epithelium. Simplified JAK-STAT signaling dynamics at anterior (pink) and posterior (blue) poles (paler colors for lower signaling and darker colors to reflect high signaling) based on the observations shown in graph F for JAK-STAT signaling intensity, and in the graph I for the number of JAK-STAT active FCs. *p*Cs are indicated in beige. St: stage. E8: Early stage 8. L8: Late stage 8. ns: non-significative. Int.: Integrated. AU: Arbitrary Units. #: Number. Statistical tests: ordinary one-way ANOVAs. Stars indicate statistically significant differences. *p < 0.05, ***p < 0.001, and ****p < 0.0001. Scale: **(B–D)** 50 µm **(G–H)** 10 µm. **(J–L)** 30 µm n: number of stage 6, 7, and 8 follicles analyzed.

JAK/STAT signaling has been highly conserved throughout evolution both structurally and functionally (Arbouzova and Zeidler, 2006). In *Drosophila*, the JAK-STAT pathway is activated upon binding of one of the three Unpaired ligands (Upd1, 2, or 3) to the unique Domeless (Dome) receptor. Following activation of the dimerized receptor, its associated kinase JAK (encoded by *hopscotch*, *hop*) phosphorylates the only STAT member (encoded by *Stat92E*), which is translocated to the nucleus to regulate target gene expression (Chen et al., 2014). Throughout oogenesis, JAK-STAT signaling plays multiple roles, requiring strict spatial and temporal regulation of its activity (Baksa et al., 2002; Beccari et al., 2002; Xi et al., 2003; Borensztejn et al., 2013). Ovarian follicles consist of a germline cyst made up of one oocyte and 15 nurse cells surrounded by a follicular monolayered epithelium. They are organized into ovarioles, which are strings of follicles progressively maturing from anterior to posterior, each connected by interfollicular stalks (Figure 1A). *upd1* (or *upd*) expression is restricted to a select group of somatic cells, polar cells (PCs), present within the follicular epithelium at anterior–posterior follicle poles (McGregor et al., 2002). Strict control of PC number, and, thus, the amount of Upd ligand, is important for JAK-STAT signaling regulation. Poles of young follicles contain groups of 3–6 PCs, and apoptosis of the supernumerary cells reduces this number to exactly 2 by stage 5 of oogenesis (Besse and Pret, 2003; Khammari et al., 2011). In this way, there is sufficient Upd early on to fulfill the JAK-STAT signaling role in interfollicular stalk formation, while by stage 7, the amount of Upd is calibrated to generate a morphogen gradient at both poles (Besse and Pret, 2003; Borensztejn et al., 2013). Consequently, three adjacent somatic cell types, border cells (BCs), stretch cells, and centripetal cells, are specified at the anterior pole, while posterior cells adopt a different fate due to the combined outputs of JAK-STAT and EGFR signaling (Xi et al., 2003; Fregoso Lomas et al., 2016; Stevens et al., 2020).

Several other processes participate in the regulation of JAK-STAT signaling in follicle cells (FCs), including Upd exocytosis and glypican-mediated diffusion between follicle and germ cells (Hayashi et al., 2012; Saadin and Starz-Gaiano, 2016), and receptor endocytosis (Devergne et al., 2007). However, only few JAK-STAT signaling regulators have been identified so far. For instance, the known repressor Socs36E, together with two transcriptional regulators (apontic and slow border cells) and *mir279* participate in the precise specification of the anterior FC fates by setting up a complex genetic circuitry acting on Stat92E and Dome (Starz-Gaiano et al., 2008; Yoon et al., 2011; Monahan and Starz-Gaiano, 2013).

As there are exactly two PCs at each follicle pole from stage 5 (Besse and Pret, 2003), these being the only source of the Upd ligand, it is understandable that JAK-STAT activity has been considered to be symmetric between anterior and posterior poles (Denef and Schüpbach, 2003; Xi et al., 2003; Hayashi et al., 2012). Some asymmetry in JAK-STAT activity has been observed between FCs located around anterior PC groups at the cell level (Manning et al., 2015), but no asymmetry has been reported at the tissue level. Here, we revisited JAK-STAT signaling pathway activity and regulation during oogenesis by using a *Stat92E* transcriptional reporter, and we observed that signaling was more dynamic than previously described. In particular, we report that JAK-STAT signaling becomes asymmetric by mid-oogenesis, with a higher activity being detected at the anterior compared to posterior poles. Interestingly, we show that the adherens junction (AJ) component Epithelial Cadherin (E-cadherin), which is involved in the formation and maintenance of epithelial structures, is also found with higher levels at the anterior follicles, leading to an asymmetry between anterior and posterior poles becoming visible at mid-oogenesis. This, together with the fact that both E-cadherin and JAK-STAT signaling are required for several morphogenesis events, including PC apoptosis as we show here, prompted us to investigate whether E-cadherin could be a regulator of JAK-STAT signaling. A key feature of AJs is their plasticity, which enables tissue remodeling. Hence, the role of E-cadherin in morphogenesis has been extensively studied in numerous tissues and models (Pannekoek et al., 2019). However, much less is known about E-cadherin acting as a regulator of cell signaling. Our novel findings demonstrate that E-cadherin is a positive regulator of JAK-STAT signaling throughout oogenesis. Finally, we found that E-cadherin and Stat92E form part of the same complex in FCs, which suggests that the regulation exerted by E-cadherin on JAK-STAT signaling could be achieved by localizing Stat92E in a permissive sub-membrane domain for efficient signaling.

## Materials and methods

### Fly stocks and genetics

The following stocks were used: *10XSTAT92E-GFP* (gift from E. Bach, Bach et al., 2007), *UAS-dome-GFP* (gift from C. Ghiglione), *shg-lacZ* (gift from JP. Vincent), *UAS-Stat92E-GFP* (gift from C. Janke); *fru-Gal4* (Borensztejn et al., 2013), *tj-Gal4* (gift from V. Mirouse), *nos-Gal4* (gift from A. Boivin), *upd-Gal4* (Khammari et al., 2011), *shg::GFP* (gift from Yang Hong); RNAi strains are from BDSC: *UAS-shgRNAi* (#38207, #32904, named #2 and #3, respectively, in the text), and VDRC: *UAS-shgRNAi* (#27082, named #1 in the text), *UAS-updRNAi* (#3282), *UAS-hopRNAi* (#102830); and *w* ^*1118*^ (#3605), Canton S (#1), *UAS-shgS* (#65589) and *tub-Gal80* ^*TS*^ (#7016) are from BDSC. *10XSTAT92E-GFP* and *shg-lacZ* were maintained at 25°C. All crosses using the Gal4-UAS system were performed at 25°C, or at 18°C when *tub-Gal80* ^*ts*^ was used (with *fru-Gal4* and *tj-Gal4* lines), until transfer of pharate pupae to 29°C. Females were dissected at 3–4 days of age.

### Immunostaining and native fluorescence

Ovary dissection and immunostaining were performed according to the work of Torres et al. (2017). For native GFP observation of *10XSTAT92E-GFP* females, ovaries were fixed in 4% formaldehyde, washed briefly 3x in PBS, 15 min in PBS-Triton 0.3%, incubated with DAPI and phalloidin for 30 min with agitation and rinsed 3x in PBS. Mounting was performed in Dabco (Sigma), and observations were carried out straightaway.

The following primary antibodies were used: rabbit anti-GFP (1:200, Interchim), rabbit anti-Stat92E (1:1000, D. Montell, used for PLA), rabbit anti-Stat92E (1:1000 S. Hou, used for immunostaining), and from DSHB: rat anti-E-cadherin (1:200), mouse anti-β-galactosidase (1:200), mouse anti-GFP (1:50), mouse anti-N-cadherin (1:200), and mouse anti-Fasciclin 3 (1:20). Secondary antibodies were all used at 1:200: anti-mouse Cy3 and anti-rat Alexa 647 (Jackson Laboratories), anti-rabbit Alexa 488, and anti-rat Alexa 647 (Thermo Fisher Scientific). Phalloidin (Sigma) and DAPI were used at 1:200 to label F-Actin and DNA, respectively.

### Image processing and analysis

PC counting and BC migration were assessed using a Leica DMRB epifluorescence microscope. Confocal images were acquired with a Leica SP8 inverted microscope driven by Las-X software, using 63x or 40x oil immersion lenses. Quantifications on confocal stacks were performed using FIJI and annotated with Affinity Designer. For GFP + FC number, we used only one confocal section passing through the middle of each follicle (visible PCs) to have consistent data regarding cell positions. For GFP quantification, total GFP signal sum projections were done. In Figure 1, measures correspond to “Raw Integrated Density” quantification of 25% follicle length at poles, whereas in Figure 3 and Supplementary Figure S4, they correspond to “Mean Grey Value” quantification of the entire follicle. All statistical analyses were performed using GraphPad PRISM.

### RT-qPCR

RT-qPCR was performed on 20 pairs of ovaries, as previously described (Parvy et al., 2012), using the following primers:

*Mirror*—5′-GAACACCGAGGATAACGATCTG-3’/5′-CGGTCATCATGTCGCCAA-3′

*H15*–5′-GGTGAAGTTGACAAACAACGAG-3’/5′-TTGGGACTGCCGGGTAT-3′

mRNA amounts were normalized with RPL49 mRNA values, used as a reference.

### Proximity ligation assay (PLA)

The Duolink PLA was performed according to the manufacturer’s instructions (Sigma-Aldrich), using mouse anti-GFP and rabbit anti-Stat92E (D. Montell) antibodies. During dissection, the muscular sheath was removed manually to allow penetration of the components in FCs, and Tween 0.1% was used instead of Triton 0.3% that we normally use for regular immunostaining.

### Co-immunoprecipitation assays

Proteins were chemically cross-linked with DSP (Thermo Fisher Scientific) prior to cell lysis. Co-immunoprecipitation assays were performed on ovary extracts from 50 females according to the manufacturer’s procedure, using GFP magnetic agarose beads (Chromotek) or Dynabeads™ protein G (Invitrogen) coated with an anti-E-cadherin antibody with 10 mM Tris-HCl pH7.5, 300 mM NaCl, 0.05% NP40 and 0.5 mM EDTA or PBS-Triton 0.02% wash buffer respectively. Proteins were resolved on NuPAGE™ 4–12% Bis-Tris gels (Invitrogen) and visualized by immunoblotting using mouse anti-GFP (1:1000; Roche) or rat anti-E-cadherin (1:250, DSHB) primary antibodies, and HRP anti-mouse (1:1000; Invitrogen) or anti-rat (1:5000; Sigma) secondary antibodies.

All experiments have been conducted between two and three times independently, and similar results were obtained in all cases.

## Results

### JAK-STAT signaling activity pattern is dynamic through oogenesis and becomes asymmetric between anterior and posterior follicle poles from stage 7 onward

In order to study JAK-STAT signaling regulation during oogenesis, we made use of a *10XSTAT92E-GFP* reporter, which drives the expression of GFP-encoding sequences in cells where the pathway is physiologically active (Bach et al., 2007). We observed that JAK-STAT activity presents a gradient in FCs with the highest activity at each follicle pole as expected, but activity did not seem identical at all stages, with a striking difference between anterior and posterior poles from mid-oogenesis, suggesting that signaling activity is finely regulated (Figures 1B–D). It is of note that we only analyzed follicles up to stage 8 since, after this stage, the anterior JAK-STAT active BCs delaminate from the follicular epithelium and migrate through nurse cells towards the oocyte, rendering analyses more difficult. We quantified the reporter activity at each pole between stages 4 and 8 using two complementary approaches: the first by determining the mean numbers of GFP-positive (GFP+) cells (Figure 1E), and the second by quantifying GFP intensities (Figure 1F). During follicle maturation, the mean GFP + FC number indicates a small reduction of the JAK-STAT gradient size at the anterior pole over time. The same tendency of fewer GFP + FCs is observed at the posterior pole from stage 7, which becomes statistically significant and more important than at the anterior pole at late stage 8, thus establishing an asymmetry in JAK-STAT signaling between poles (Figure 1E). In addition, while the gradient size decreases, there is an increase in signaling intensity at both poles between stages 7 and 8, but signaling intensity was significantly lower at the posterior pole (Figure 1F). Both techniques thus confirmed that JAK-STAT signaling activity is symmetrical at both poles up to stage 7 but becomes asymmetrical at stage 8. Our results, therefore, uncover an asymmetry of JAK-STAT signaling in the number and intensity of active cells between anterior and posterior poles.

To verify this result, we tested another readout for JAK-STAT signaling. While in some cases the JAK-STAT receptor is active when it is localized at the plasma membrane (Blouin et al., 2016), receptor endocytosis can sometimes be a requirement for signaling activity (Marchetti et al., 2006). In all cases, receptor endocytosis reflects active transduction. In the follicular epithelium, the accumulation of Dome-GFP vesicles has been described as an indicator of efficient endocytosis, thus being an indicator of JAK-STAT activity (Silver et al., 2005; Devergne et al., 2007; Sahu et al., 2017). We expressed a GFP-fused form of Dome in all FCs with the *fruitless-Gal4 (fru-Gal4)* driver (Supplementary Figure S1A for driver pattern) and assessed JAK-STAT signaling by counting the number of cells containing Dome-positive vesicles (Figures 1G–I). A progressive decrease in mean numbers of FCs containing Dome vesicles is observed at both poles, confirming that JAK-STAT gradient size decreases with time. Consistently with our previous results, the decrease is smaller at the anterior pole than at the posterior pole, creating an asymmetry in JAK-STAT signaling between poles. There is a slight lag between the translation of the signaling asymmetry into a visible output between the two reporters, the asymmetry being visible with Dome endocytosis from stage 7 and with *10XSTAT92E-GFP* from stage 8, reflecting an expected difference in the kinetics between these two types of reporters.

Taken together, and as schematized in Figure 1M, our findings demonstrate that, although anterior and posterior follicle poles display similar levels of JAK-STAT signaling activity up to stage 6, fewer posterior than anterior FCs are active as of stage 7. This creates an asymmetry between poles in the JAK-STAT signaling gradient size and intensity that must be precisely regulated.

### E-cadherin expression pattern is dynamic through oogenesis and becomes asymmetric between anterior and posterior follicle poles from stage 6 onward

Upd, secreted from PCs at each follicle pole, activates the JAK-STAT pathway in surrounding FCs. At stage 7, when we showed signaling becomes asymmetric, PCs have reached their final number of two at each pole. Hence, the asymmetry in JAK-STAT signaling activity cannot be explained by a difference in the number of ligand secreting cells. Rather, we hypothesized that a regulator could be acting within the follicular epithelium to control JAK-STAT signaling activity. In that case, the regulator should display similar dynamics in its expression, with an asymmetry between poles from stage 7 or slightly earlier to allow time for the JAK-STAT pathway to become asymmetric in response to its regulation. We tested several proteins that might respond to such criteria and found E-cadherin as a good candidate. During oogenesis, as reported previously (Godt and Tepass, 1998), E-cadherin is present in all follicle and germline cells, which can be observed with an E-cadherin immunostaining (Figures 1J,K). At the subcellular level, E-cadherin is detected differentially along the apico-basal axis: strong levels are observed at the apical membrane, where FCs not only interact together but also with germ cells (GCs), which represents an unusual E-Cadherin-mediated kind of adhesion as it occurs between different cell types (Godt and Tepass, 1998; González-Reyes and St Johnston, 1998), whereas lower levels are detected at the lateral AJs where FCs only interact with other FCs (Figure 1J). At the tissue level, although E-cadherin is distributed homogenously along the anterior-posterior axis until stage 5, stronger accumulation is observed in anterior compared to posterior FCs, the difference being accentuated at stages 7 and 8, while follicle size is increasing (Figures 1J,K). This result shows that E-cadherin distribution in FCs becomes asymmetric between anterior and posterior follicle regions from stage 6.

To determine whether the asymmetric distribution of E-cadherin in FCs along the anterior-posterior axis is due to post-translational modifications, affecting for instance protein stability or recycling, or if asymmetry is rather regulated at the transcriptional level, we used a transcriptional reporter (*shotgun-lacZ*). In this line, β-galactosidase detection reflects the activity of regulatory sequences of *shotgun* (*shg*), the gene encoding the E-cadherin protein in *Drosophila*. We found homogenous expression of the *shg-lacZ* reporter in FCs until stage 5, which becomes differential between anterior and posterior regions at stage 6, except for expression in PCs that are maintained high at all stages (Figure 1L). This demonstrates that the E-cadherin asymmetry in FCs between anterior and posterior follicle regions being set at stage 6 is regulated at the transcriptional level.

Altogether our findings show that JAK-STAT activity and *shg* expression display similarities in their spatial and temporal dynamics in the follicular epithelium during oogenesis. Notably, by mid-oogenesis, both E-cadherin levels and JAK-STAT activity are high in the anterior follicles, while they are significantly lower in the posterior (Figure 1M), raising the hypothesis that they could be involved in similar processes.

### E-cadherin, like JAK-STAT signaling, is necessary for PC apoptosis

We, thus, wondered if E-cadherin and JAK-STAT signaling correlate in function during oogenesis. We investigated this hypothesis by using RNAi lines targeting *shg* and *upd*. To assess whether our tools were efficient, we first tested if we could observe known phenotypes associated with *shg* and *upd* knock-downs with these RNAi lines. It has been independently shown that E-cadherin (Niewiadomska et al., 1999; Pacquelet and Rørth, 2005) and JAK-STAT signaling (Beccari et al., 2002; Silver et al., 2005) are involved in BC migration, a cellular process occurring between stage 9 and 10. In addition, a genetic interaction between *stat92E* and *shg* was reported in BC migration (Silver and Montell, 2001). We used the *upd*-*Gal4* driver to target RNAi against *upd* specifically in PCs (Supplementary Figure S1B for driver pattern**)**, thus decreasing JAK-STAT signaling in FCs from stage 2 onward. We assessed BC migration in the resulting follicles at stage 10, when the migration process is complete in controls. 97% of follicles exhibit BC migration delay in an *upd* knock-down context (Supplementary Figure S2B,E). We next used two RNAi lines against *shg,* which we showed were efficient at decreasing E-cadherin levels in FCs (#1) or in GCs (#2) when driven with the *fru-Gal4* or the *nos*-Gal4 lines respectively (Supplementary Figure S1A,B for driver patterns), as revealed by the absence of E-cadherin signal detection in the corresponding tissue (Supplementary Figure S2C,D). In both cases, we observed BC migration defects in more than 90% of stage 10 follicles (Supplementary Figure S2C–E). The RNAi tools with the Gal4 lines used are therefore efficient to affect JAK-STAT signaling and E-cadherin functions, leading to the expected BC migration delay phenotypes.

**FIGURE 2:**
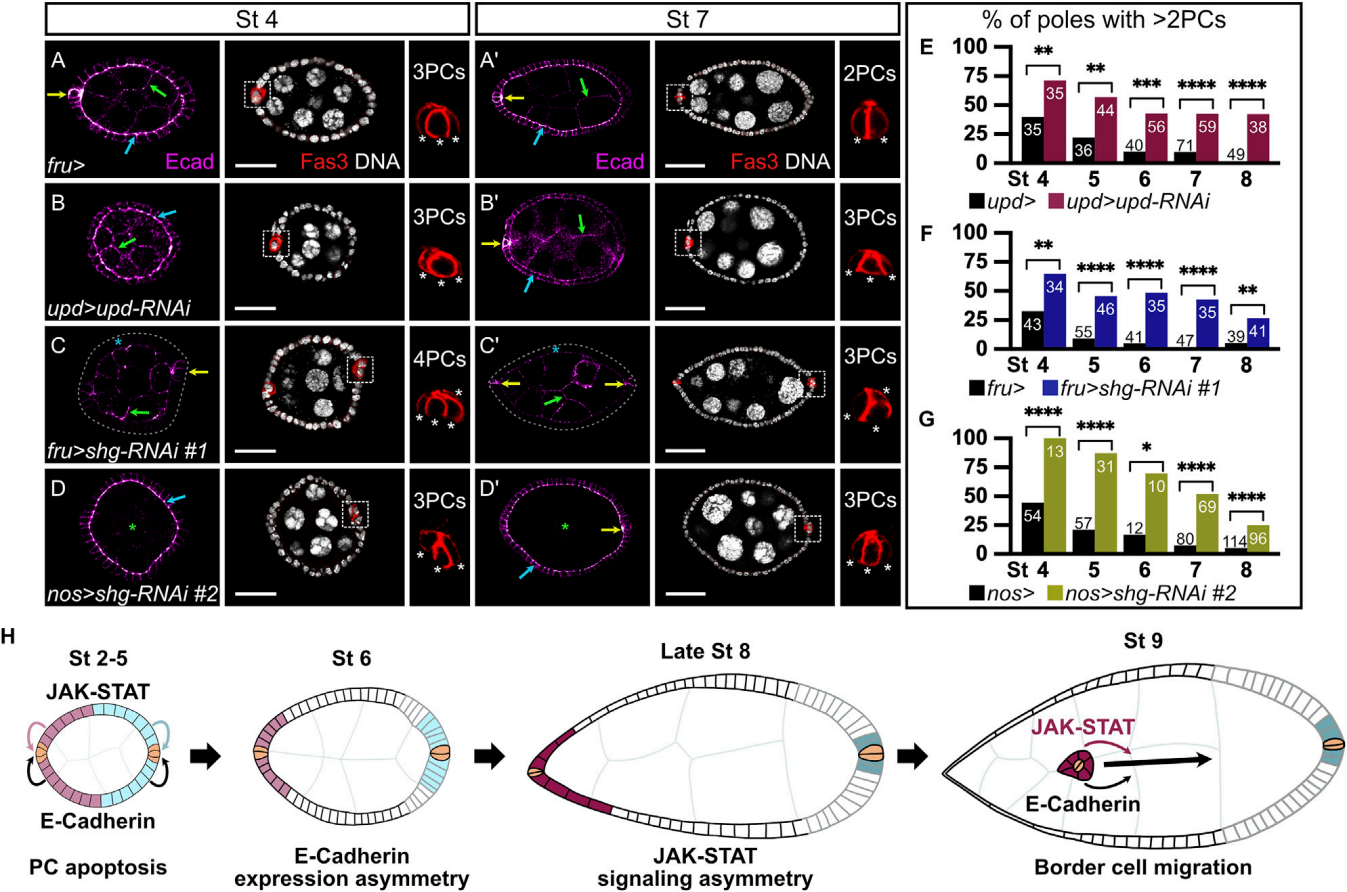
Somatic and germinal E-cadherin is necessary for JAK-STAT-mediated polar cell apoptosis. In all images and drawings, the anterior is to the left, except for polar cell (PC) magnifications for which apical side is up. Follicles at stages 4 **(A–D)** and 7 **(A′–D′)** of oogenesis are shown. E-cadherin (Ecad, “magenta hot” filter, from magenta, low intensity, to white, high intensity) and the PC marker Fasciclin3 (Fas3, red) are detected by immunostainings and DNA by DAPI (grey). E-cadherin detection is indicated in follicle cells (FCs) by blue arrows, in *p*Cs by yellow arrows, and in germline cells by green arrows. To assess the efficiency of PC apoptosis after stage 5 of oogenesis, we used Fas3 staining to count PC numbers at various stages. In the images with Fas3 and DAPI stainings, each white dotted box highlights one PC group, which is magnified to the right to depict how *p*Cs are counted (each white asterisk indicates one PC). PCs at the opposite follicle pole are not always visible on the displayed z sections. **(A,A′)** Follicles from females carrying the *fru-Gal4* driver, as a control for PC apoptosis. Although supernumerary *p*Cs can still be detected at stage 4 **(A)**, only 2 are normally found at stage 7 **(A′)**. E-cadherin is detected in PCs, in germline cells and apico-laterally in FCs. **(B,B′)** Follicles from females expressing RNAi targeting *upd* in *p*Cs driven by *upd-Gal4*. These follicles present a defect in PC apoptosis, as illustrated by the presence of supernumerary *p*Cs at stage 7 **(B′)**. **(C,C′)** Follicles from females expressing the RNAi line #1 targeting *shg* driven by *fru-Gal4* to knock-down *shg* in FCs, except *p*Cs where *fru-Gal4* is not expressed. E-cadherin is not detected in FCs (blue asterisk), but is still detected in the germline and in PCs. Gray dotted line delimits the basal side of the follicular epithelium. The presence of supernumerary *p*Cs at stage 7 **(C′)** indicates a defect in the PC apoptosis process. **(D,D′)** Follicles from females expressing the RNAi line #2 targeting *shg* with the germline specific *nos-Gal4* driver, showing that E-cadherin is not detected in germ cells (green asterisk), but is maintained in the follicular epithelium and PCs. The presence of supernumerary *p*Cs at stage 7 **(D′)** indicates a defect in the PC apoptosis process. **(E–G)** Apoptosis defect quantification illustrated by the percentage of poles with more than 2 PCs at various stages of oogenesis when *upd* is knocked down in PCs (E, pink bars), and when *shg* is knocked down in FCs (F, blue bars) or in germ cells (G, green bars) compared to control conditions (females carrying only the corresponding *Gal4* drivers, black bars). Numbers in bars correspond to the number of counted poles. As in the JAK-STAT knock-down condition, a significant perturbation in PC apoptosis is observed upon somatic and germinal *shg* knock-down at all stages of oogenesis analyzed. **(H)** Schematic drawing representing the E-cadherin expression pattern (black and gray lines for high and low E-cadherin, respectively), together with JAK-STAT signaling dynamics (pink and blue-filled FCs, same color code as presented in Figure 1) in the follicular epithelium and recapitulating JAK-STAT and E-cadherin shared roles at different stages of oogenesis: for PC apoptosis between stages 2 and 5 and for border cell migration at stage 9. *p*Cs are indicated in beige. St: stage. Statistical tests: Chi2. Stars indicate statistically significant differences. *p < 0.05, **p < 0.01, ***p < 0.001, and ****p < 0.0001. Scale: (St 4) 20 µm, (St 7) 30 µm.

We, thus, used the same tools to investigate whether E-cadherin expression and JAK-STAT signaling are also involved in another common process during oogenesis. PC apoptosis takes place between stages 2 and 5 of oogenesis, resulting in only two PCs at each pole (Besse and Pret, 2003, Figure 2A). JAK-STAT signaling has been shown to regulate morphogenesis leading to PC apoptosis by monosis in a non-cell-autonomous manner (Borensztejn et al., 2013; Torres et al., 2017, Figures 2B,E). We knocked down *shg* using the *fru-Gal4* driver, which results in the absence of E-cadherin staining in FCs, without disturbing epithelial integrity as N-cadherin is also expressed in FCs thereby ensuring the maintenance of the follicular epithelium even in the absence of E-cadherin (Supplementary Figure S3A,B). We then assessed whether completion of PC apoptosis occurred by counting the number of PCs at each pole from stages 4–8. An excess of PCs (>2 per group at stage 7) was observed in the *shg* knock-down, with a significant difference from the control at all stages, reflecting a defect in the apoptotic process (Figures 2C,F). Similar results were obtained using yet another RNAi line against *shg* (#3) with a second FC driver (*traffic jam-Gal4*, *tj-Gal4*) (Supplementary Figure S1D for driver pattern and Supplementary Figure S4A). E-Cadherin, like the JAK-STAT pathway, is therefore required non-cell-autonomously in FCs for correct PC apoptosis.

E-cadherin can be found apically in FCs, at the interface with GCs (FC-GC contact) and laterally, at the interface between FCs (at FC-FC AJs only). To gain more insight as to which FC interface is involved in PC apoptotic extrusion, we decreased E-cadherin levels in GCs by knocking down *shg* from the germline with the RNAi line #2 driven by the *nos*-*Gal4* driver, which is expected to disturb junctions at the FC-GC interface. As with the somatic *shg* knock-down, epithelial integrity is maintained in the germline knock-down (Supplementary Figure S3C), and extra PCs, indicative of apoptosis defects, are observed with significant differences at all stages compared to controls (Figures 2D,G). Our data thus show that decreasing E-cadherin in FCs or in GCs disturbs PC apoptosis in a non-cell-autonomous manner, similarly to decreasing JAK-STAT pathway activity in the follicular epithelium. Hence, E-cadherin and JAK-STAT signaling are both involved in two distinct morphogenesis processes that occur at different stages of oogenesis, namely PC apoptotic extrusion and BC migration, as schematized in Figure 2H. Altogether, our data demonstrate that E-cadherin accumulation and JAK-STAT signaling partially overlap in dynamics and share functions during oogenesis.

### E-cadherin regulates JAK-STAT activity positively in the follicular epithelium during oogenesis

**FIGURE 3:**
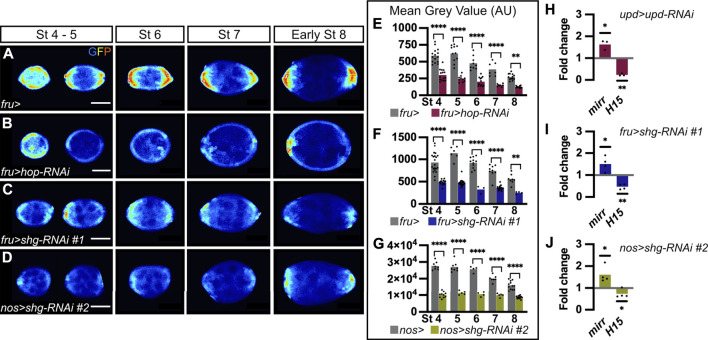
E-cadherin is a positive regulator of JAK-STAT signaling. In all images, the anterior is to the left. **(A–D)** Follicles at various stages of oogenesis from *10XSTAT92E-GFP* hemizygous females in four different genetic backgrounds. GFP immunostaining (“royal” filter, sum projection of total GFP signal) reflects JAK-STAT activity at each pole. **(A)** Follicles from females carrying the *fru-Gal4* driver are used as a control to assess JAK-STAT activity, which forms a gradient in follicle cells (FCs) at each pole throughout oogenesis. **(B)** Follicles from females expressing a RNAi targeting *hop* in FCs driven by *fru-Gal4* to decrease JAK-STAT signaling, as illustrated by weak GFP signal at each pole from stage 4 onward. Follicles from females expressing the RNAi line #1 targeting *shg* driven by *fru-Gal4* in FCs **(C)** or the RNAi line #2 also targeting *shg* but driven in germ cells (GCs) by *nos*-*Gal4* **(D)**. In both knock-down conditions, GFP signal is low in the entire follicular epithelium from stage 4 onward. **(E–G)** Mean Grey Value quantifications of entire follicles at various stages of oogenesis. In graphs, bars represent mean measurements and each dot corresponds to one measurement. Quantifications were made on sum projections of total GFP signal upon knock-down of *hop* (E, pink bars) and *shg* in FCs (F, blue bars), and *shg* in GCs (G, green bars), compared to control conditions (females carrying the corresponding Gal4 drivers, grey bars). As for JAK-STAT pathway inhibition, GFP intensity is significantly lower than in controls upon somatic and germinal *shg* knock-down at all stages analyzed. In graph G, the mean gray value scale is higher because images were taken with higher bit depth and pixel size than for graphs E-F. **(H–J)** RT-qPCR analysis of *mirror* (*mirr*) and *H15* transcript abundance, negative and positive targets, respectively, of the JAK-STAT pathway. In graphs, bars represent mean measurements from independent experiments and each dot corresponds to one independent experiment. Processed RNAs were extracted from entire ovaries of females expressing RNAi targeting *upd* in polar cells (H, *upd-Gal4*), *shg* in FCs (I, *fru-Gal4*) and *shg* in GCs (J, *nos-Gal4*). Expression of both transcripts are normalized with *RPL49* expression and compared to relative expression in control conditions (females carrying the corresponding Gal4 drivers). As upon JAK-STAT signaling inhibition (H, *upd > upd-RNAi*), *mirr* and *H15* relative expressions increase and decrease, respectively, upon somatic and germinal *shg* knock-down (I, *fru > shg-RNAi* and J, *nos > shg-RNAi*, respectively), indicating a decrease in JAK-STAT signaling under both conditions. St: stage. Statistical tests: **(E–G)** Ordinary one-way ANOVAs **(H–J)** One-tailed ratio paired t-test. Stars indicate statistically significant differences. *p < 0.05, **p < 0.01 and ****p < 0.0001. Scale: 30 µm.

As E-cadherin is distributed in a larger domain than the one covered by JAK-STAT activity, and given that E-cadherin asymmetry between poles starts at stage 6, just before the asymmetry in JAK-STAT activity is visible, at stage 7, we wondered whether E-cadherin could be a general regulator of JAK-STAT signaling in the follicular epithelium. To test this hypothesis, we decreased E-cadherin levels by knocking down *shg* in FCs, in flies carrying the *10XSTAT92E-GFP* transcriptional reporter, which allows for the assessment of JAK-STAT activity levels. As a control, we first decreased JAK-STAT activity by targeting RNAi against *hop*, encoding the kinase of the pathway, in FCs. As expected, when *hop* was knocked down, the JAK-STAT pathway activity reporter was strongly reduced at all stages, compared to controls (Figures 3A,B). Mean GFP intensity was quantified on sum projections of total GFP in the whole follicular epithelium, and, accordingly, a significant decrease was found at all stages considered (Figure 3E). When E-cadherin levels were decreased in FCs by knocking down *shg* (RNAi line #1), similar results were obtained, with the *10XSTAT92E-GFP* reporter activity being reduced in the whole epithelium and mean GFP intensity being significantly decreased at all stages analyzed as compared to controls (Figures 3C,F). We confirmed that knocking down *shg* in FCs results in reduced JAK-STAT activity by expressing the *shg* RNA line #3 with *tj-Gal4* (Supplementary Figure S1D for driver pattern and Supplementary Figures S4B–D).

As we found that E-cadherin is required in FCs and GCs to regulate PC apoptosis, we wondered if both FC and GC E-cadherin were also involved in the regulation of JAK-STAT signaling. We thus knocked down *shg* in the germline with the *nos-Gal4* driver. Again, we found the JAK-STAT transcriptional reporter activity reduced in the whole follicular epithelium, with mean GFP intensity being significantly decreased at all stages compared to control (Figures 3D,G). This suggests that E-cadherin is required at FC-FC and FC-GC interfaces to regulate JAK-STAT activity in the follicular epithelium. In summary, our findings, which were confirmed with three different RNAi lines and three different Gal4 drivers, demonstrate that *shg* expression is required in FCs and in GCs for proper JAK-STAT activity in the follicular epithelium, suggesting that E-cadherin is a positive regulator of JAK-STAT signaling during oogenesis.

To confirm these findings by monitoring JAK-STAT pathway activity in a different way, we quantified the expression of JAK-STAT known target genes by RT-qPCR. *mirror (mirr)* is negatively regulated in response to JAK-STAT signaling (Xi et al., 2003), whereas *H15* is positively activated by the pathway (Fregoso Lomas et al., 2016). Accordingly, we found that *mirr* was upregulated and *H15* downregulated in ovarioles in which RNAi was used to knock-down *upd* in PCs, thus decreasing JAK-STAT signaling in the follicular epithelium (Figure 3H). When E-cadherin levels were decreased by knocking down *shg* in FCs or in GCs, similar *mirr* upregulation and *H15* downregulation were obtained (Figures 3I,J)*.* Given that similar changes in the expression of JAK-STAT target genes are detected upon decreasing JAK-STAT signaling and E-cadherin levels, this further confirm that E-cadherin is a positive regulator of JAK-STAT signaling during oogenesis.

### E-cadherin and Stat92E are part of a physical complex in the follicular epithelium during oogenesis

**FIGURE 4:**
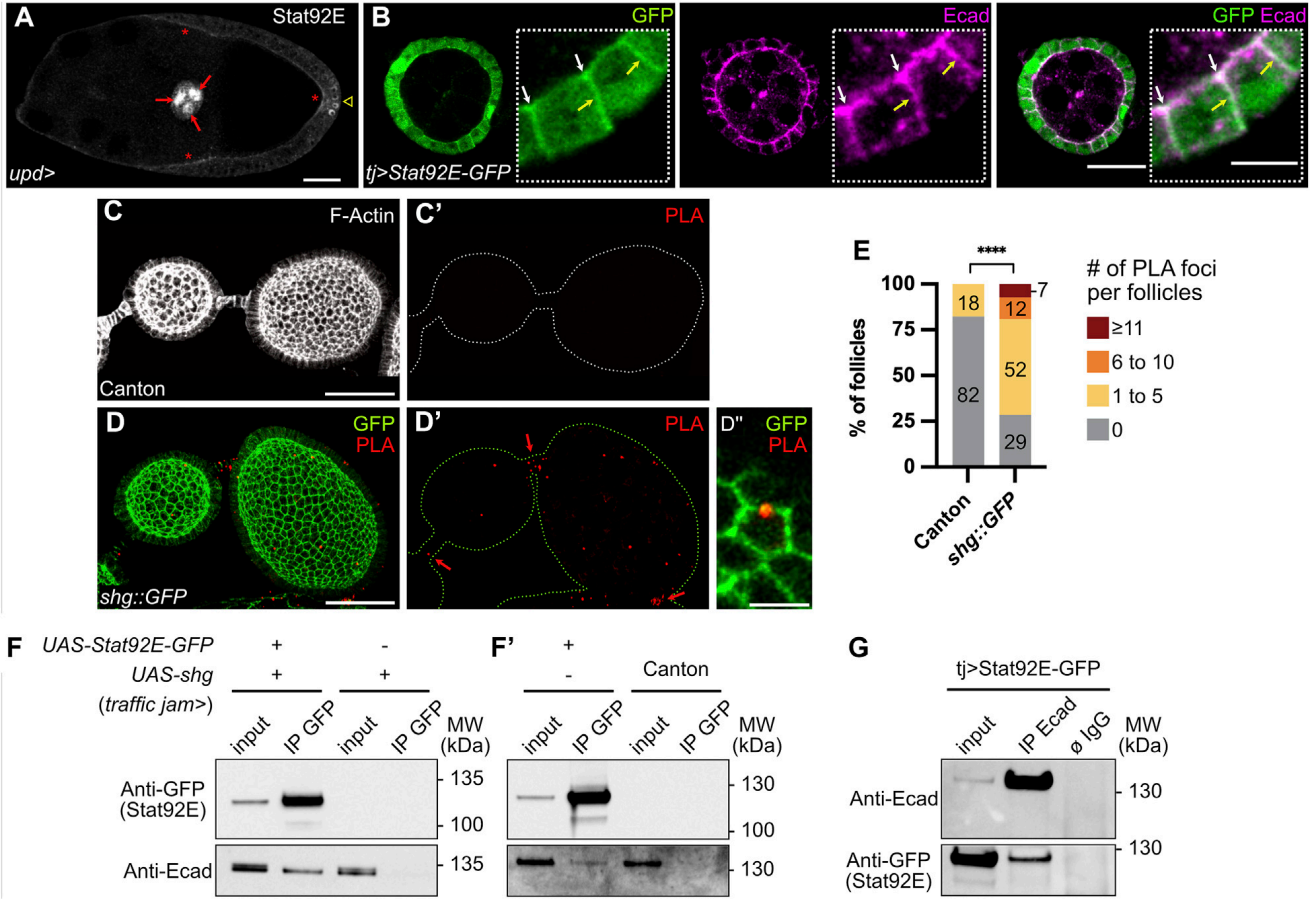
E-cadherin and Stat92E are part of a physical complex in the follicular epithelium. In all images, the anterior is to the left. **(A)** Stage 9 follicle of a control female (*upd-Gal4/+)* in which the transcription factor Stat92E is immunodetected (grey) on the apical side of follicle cells (FCs, red stars). Nuclear Stat92E is also detected at high levels in border cells (red arrows) and at the posterior pole, where the JAK-STAT pathway is highly active. Sum z projection of 5 confocal sections. **(B)** Stage 4 follicle from a female overexpressing *UAS-Stat92E-GFP* in FCs driven by *traffic jam-Gal4* (*tj*). Immunostainings of GFP (green) and E-cadherin (Ecad, magenta) on one confocal section show that both Stat92E-GFP and E-cadherin have localized apically (white arrows) and laterally (yellow arrows) and that both signals coincide partially at apical and lateral junctions. **(C,D)** Proximity Ligation Assay (PLA) performed with an anti-Stat92E antibody and an anti-GFP antibody on ovaries from Canton females, used as negative controls **(C)** and females bearing a *shg::GFP* construct (Knock-In, **(D)**. Images are max projections of all confocal sections except for the magnification **(D″)**, presenting one confocal section. **(C,C′)** One representative control follicle in which no PLA foci are detected. F-actin staining (phalloidin, grey) allows to visualize FCs. **(D,D′)** PLA foci (red) are detected in *shg::GFP* follicles and stalk cells (red arrows). Native GFP signal (green) shows that PLA foci are in close proximity with E-cadherin:GFP rich junctions in FCs (see magnification in D″). **(E)** Quantification of PLA foci in Canton and *shg::GFP* follicles. PLA foci were counted on each z section, and total foci numbers per follicle were classified into four categories: 0 focus; 1 to 5 foci; 6 to 10 foci; 11 and more foci. In the graph, the size and the numbers within the bars indicate the percentage of follicles in each category showing that the number of follicles with PLA foci is significantly higher in *shg::GFP* flies than in Canton flies. PLA foci in stalk cells were not quantified **(F,F′,G)** Western blots showing results of immunoprecipitations (IP) on total ovary protein extracts (input) from females expressing or not *UAS-Stat92E-GFP* and/or *UAS-shg* in FCs driven by *tj-Gal4*. Blots were probed with anti-GFP or anti-Ecad antibodies. **(F,F′)** IP using GFP-trap beads. The extracts from *shg* overexpression condition and the canton extract are negative controls showing that E-cadherin does not, in the absence of Stat92E-GFP, bind to GFP-trap beads (lanes 4). Overexpressed (F, lane 2) and endogenous (F′, lane2) E-cadherin proteins are co-immunoprecipitated with Stat92E-GFP, showing E-cadherin and Stat92E-GFP are part of a physical complex. **(G)** Reverse IP using anti-Ecad coated beads. The non-coated beads condition (ø IgG) is a negative control showing that neither E-cadherin nor Stat92E-GFP binds specifically to the beads. Stat92E-GFP is co-immunoprecipitated with endogenous E-cadherin (lane 2), confirming that E-cadherin and Stat92E-GFP are part of a physical complex. #: Number. MW: Molecular Weight. Statistical tests: **(E)** Fisher test comparing the number of follicles with 0 and with at least 1 PLA foci. Number of stage 2–8 follicles analyzed: Canton *n* = 45, *shg::GFP* n = 42. Stars indicate statistically significant differences. ****p < 0.0001. Scale: **(A,C,D)** 30 µm **(B)** 20 µm. All magnifications: 5 µm.

The importance of Stat92E localization for efficient signaling activity has been shown in the embryo ectoderm epithelium, where its apical localization is ensured by interaction with the polarity protein Bazooka (Baz), a *Drosophila* Par3 homolog (Sotillos et al., 2008). To find out if the apical localization of Stat92E is conserved in ovaries, we looked at control follicles stained with an anti-Stat92E antibody. Besides the expected nuclear staining at follicle poles and in border cells at stage 9, where JAK-STAT activity is the highest, we also detected endogenous Stat92E apically in FCs (Figure 4A). Similarly, when we overexpressed a GFP-tagged version of Stat92E, besides the enrichment in nuclei at follicle poles, Stat92E was also localized apically and laterally in FCs from stage 3 to stage 9, partially overlapping with E-cadherin (Figure 4B). To gain more insight into the molecular mechanism by which E-cadherin could regulate JAK-STAT activity in follicles, we tested whether E-cadherin and Stat92E were in close proximity in FCs by performing a proximity ligation assay (PLA). For this, we used anti-Stat92E and anti-GFP antibodies on ovaries from *shg::GFP* Knock-In flies to test for E-cadherin-GFP/Stat92E proximity. Although it is not easy to detect PLA signals in ovaries due to penetration issues (Mannix et al., 2019), we found a significantly higher number of follicles with PLA foci in *shg::GFP* ovaries, as compared to the negative control that was nearly devoid of PLA signal (Figures 4C–E), suggesting that E-cadherin and Stat92E can be found in close proximity in FCs.

Finally, we tested whether E-cadherin and Stat92E could interact together in ovaries by performing co-immunoprecipitation assays. For this, *Stat92E*-*GFP* and *shg* were overexpressed in FCs with the *tj*-*Gal4* driver, and co-immunoprecipitation was tested by using beads bound with anti-GFP antibodies (Figure 4F,F′). When only *shg* is overexpressed, E-cadherin is not immunoprecipitated from ovary extracts incubated with anti-GFP beads (Figure 4F, lane 4). However, when both *Stat92E-GFP* and *shg* are co-overexpressed, E-cadherin is co-immunoprecipitated with Stat92E-GFP from ovary extracts incubated with anti-GFP beads (Figure 4F, lane 2). Endogenous E-cadherin was also co-immunoprecipitated from flies expressing only Stat92E-GFP in FCs (Figure 4F’, lane 2**)**. To confirm these results, we also performed the reverse immunoprecipitation on flies expressing only Stat92E-GFP in FCs and succeeded in co-immunoprecipitating Stat92E-GFP from E-Cadherin-coated beads, whereas no Stat92E was immunoprecipitated with the beads alone (Figure 4G). Our results indicate that Stat92E-GFP and endogenous E-cadherin are part of the same physical complex in FCs.

Altogether, our study unravels that E-cadherin is a new positive regulator of the JAK-STAT pathway during oogenesis and that E-cadherin and Stat92E are part of a complex in FCs, suggesting that E-cadherin could modulate JAK-STAT signaling in these cells through physical binding to Stat92E.

## Discussion

The results presented here reveal that E-cadherin is necessary for optimal JAK-STAT signaling in the *Drosophila* follicular epithelium during oogenesis. They also show that *shg* is expressed differentially at follicle poles, leading to lower E-cadherin accumulation in the posterior region from mid-oogenesis, compared to the anterior region, and that JAK-STAT signaling displays a similar asymmetry. The decrease in E-cadherin levels at the posterior pole is therefore likely responsible for the concurrent decrease in JAK-STAT signaling. It will be interesting in the future to elucidate whether the asymmetry in anterior-posterior JAK-STAT signaling in follicles has a functional significance in their development.

Our study also shows that E-cadherin is necessary for both FCs and adjacent GCs for full JAK-STAT activation in the follicular epithelium in proximity to ligand-producing PCs at follicle extremities. This implies that it is the role of E-cadherin in cell–cell contact, including at the FC-GC interface, that is important for high levels of JAK-STAT signaling. In addition, our work demonstrates that Stat92E is in a complex with E-cadherin in FCs. These results provide potential mechanistic models for the role of E-cadherin in activating JAK-STAT signaling. Indeed, the importance of JAK-STAT components subcellular localization has been demonstrated in FCs, as localization of *upd* mRNA, Upd secretion (Van de Bor et al., 2011) and Dome endocytosis (Devergne et al., 2007) all occur apically. Since we found some Stat92E localized apically in FCs and since the majority of E-cadherin is also detected apically in these cells, it is possible that the E-cadherin-Stat92E interaction allows Stat92E accumulation in proximity to the apically-localized Dome receptor. This polarization of Stat92E in a permissive subdomain of the plasma membrane may mediate Dome conformational change or stabilize the Dome-Stat92E complex at the membrane, leading to more efficient signaling. Similar mechanisms have been demonstrated in mammals, such as in human fibroblasts, where IFN-γR localization in either membrane lipid or actin nanodomains can have, respectively, an inhibiting or a permissive action on JAK activation, by regulating a conformational change in the receptor (Blouin et al., 2016).

Precedent for polarized JAK-STAT signaling also exists in *Drosophila* since, in the embryonic ectoderm epithelium, apical receptor localization dictates signal transduction (Hombría and Sotillos, 2008). In these cells, subapical accumulation of cytoplasmic inactive Stat92E depends on Par-3 (Sotillos et al., 2008), their physical interaction being mediated by Src kinases (Sotillos et al., 2013). Therefore, polarized apical JAK-STAT signaling not only in the embryonic ectoderm but also in the follicular epithelium (our present findings) may involve interactions with the transmembrane proteins Par-3 and E-cadherin, respectively. Our findings, thus, extend the phenomenon of polarized apical JAK-STAT signaling to a second epithelial tissue in *Drosophila*, the follicular epithelium.

It is possible that Par-3 also associates with the E-cadherin-Stat92E complex, which could contribute to apically polarized JAK-STAT signaling in ovaries. Indeed, Par-3 is physically associated with AJs in different species (Coopman and Djiane, 2016; Thompson, 2022), and Stat92E interacts with Par-3 in the *Drosophila* embryo (Sotillos et al., 2008). Consequently, it has been proposed, although it was not demonstrated, that, generally, Par-3 may form a bridge between E-cadherin and Stat92E, which retains Stat92E at the plasma membrane (Ramirez Moreno et al., 2021).

Finally, E-cadherin could also regulate JAK-STAT signaling outside of the ovary as both E-cadherin and JAK-STAT are involved in other common processes, such as germline stem cell maintenance in *Drosophila* testis (Wang et al., 2006). Although JAK-STAT signaling regulation by E-cadherin has not been described in *Drosophila* before the present study, in mammals, cadherin activation of JAK-STAT signaling has been reported in several systems. In rat testis, the short-type PB-cadherin, a classical cadherin that co-immunoprecipitates with JAK2, is necessary for JAK-STAT activation and GC survival (Wu et al., 2005). In mouse embryonic stem cells, E-cadherin association with the LIFR-GP130 receptor complex and its consequent stabilization are important to maintain JAK-STAT signaling and pluripotency (del Valle et al., 2013). In cultures of a squamous carcinoma cell line, E-cadherin and STAT3 colocalize and E-cadherin-based cell–cell contacts are necessary for STAT3 activity (Onishi et al., 2008; Geletu et al., 2013). Our findings are, thus, particularly exciting since they suggest conservation of JAK-STAT regulation by E-cadherin between mammals and *Drosophila* and, therefore, strengthen the use of the *Drosophila* follicular epithelium as an excellent model to study JAK-STAT pathway regulation.

## Data Availability

The original contributions presented in the study are included in the article/Supplementary Material; further inquiries can be directed to the corresponding author.

## Abbreviations

AJ, adherens junction; BC, border cell; GC, germ cell; FC, follicle cell; PC, polar cell; *shg*, *shotgun; upd*, *unpaired; hop*, *hopscotch; fru*, *fruitless; nos, nanos; tj*, *traffic jam; mirr*, *mirror; baz*, *bazooka*.
