# Supplementary figures and images for: E-cadherin acts as a positive regulator of the JAK-STAT signaling pathway during Drosophila oogenesis
Source: Front Cell Dev Biol. 2022 Aug 23;10:886312. doi: 10.3389/fcell.2022.886312 (PMC9473917; doi:10.3389/fcell.2022.886312)

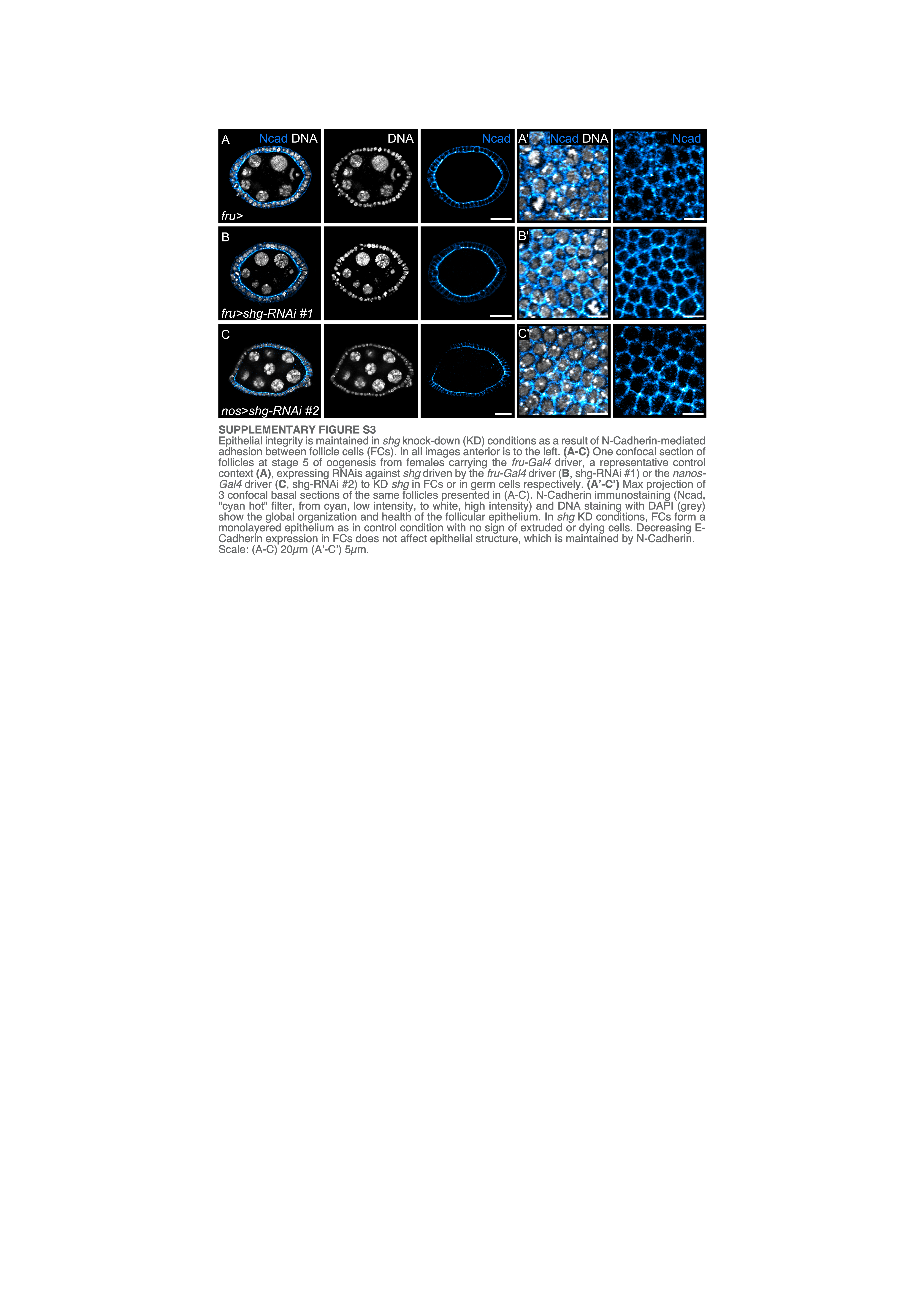

Supplement: Supplementary file 1 [file Image3.tiff]

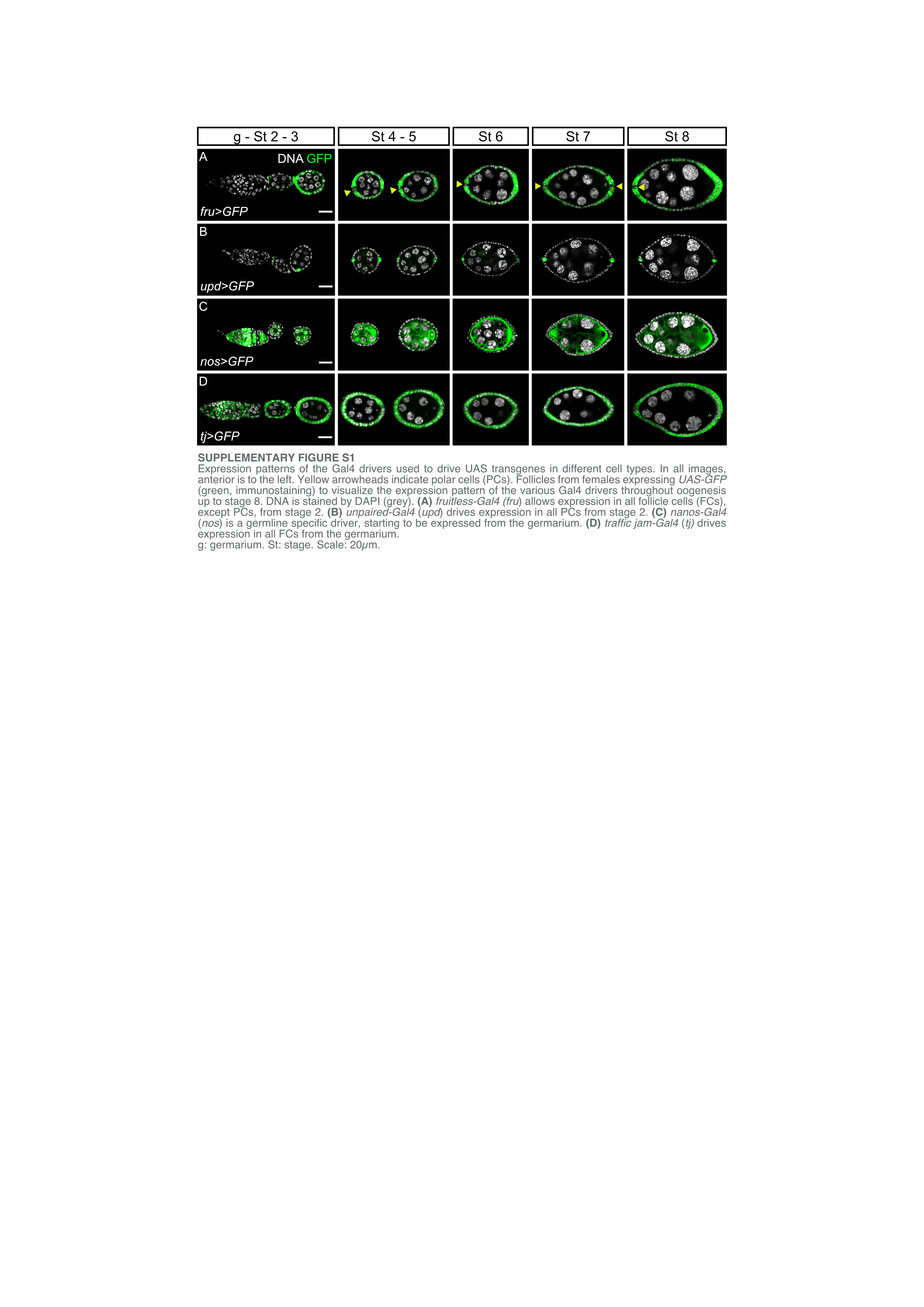

Supplement: Supplementary file 2 [file Image1.tiff]

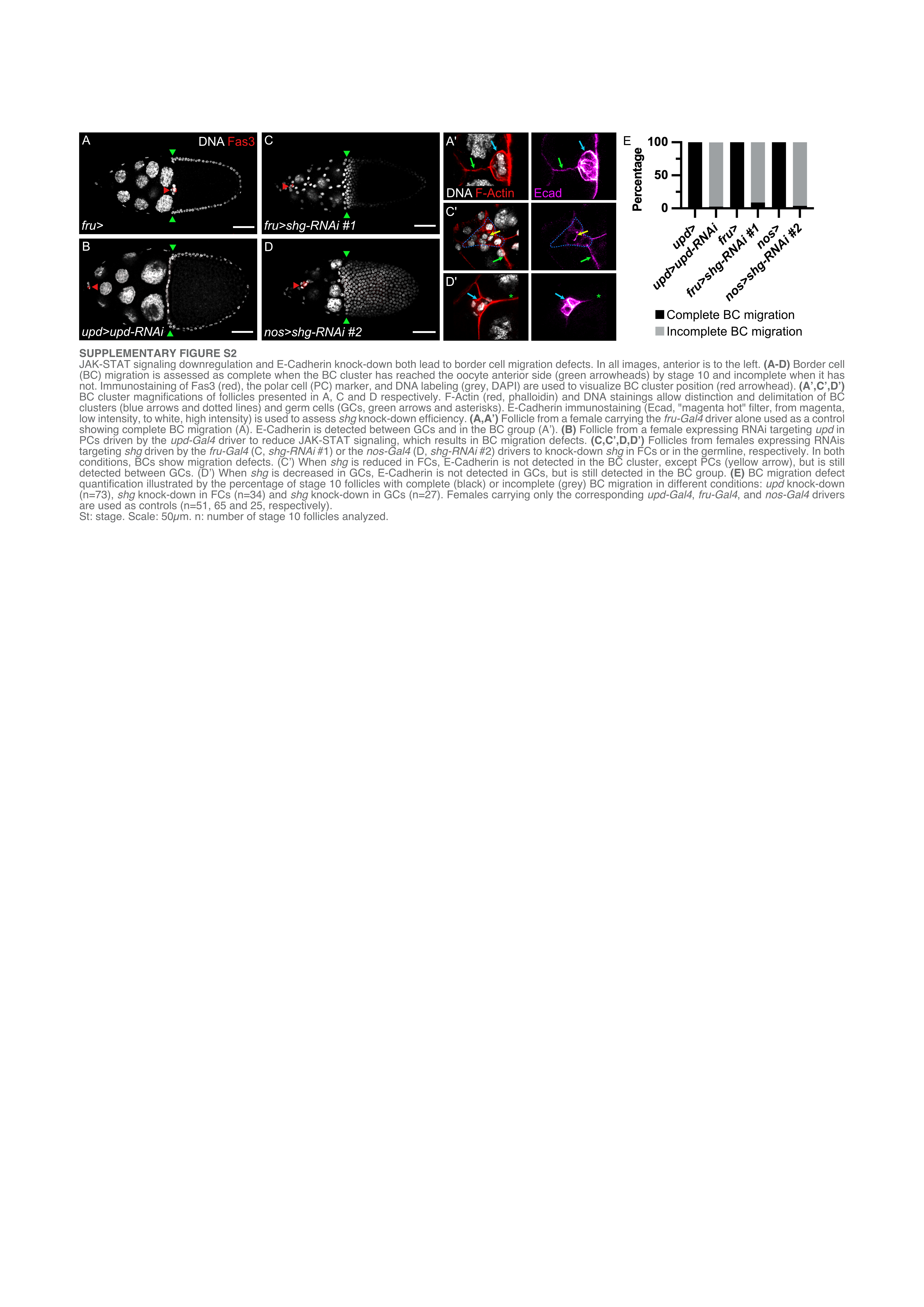

Supplement: Supplementary file 3 [file Image2.tiff]

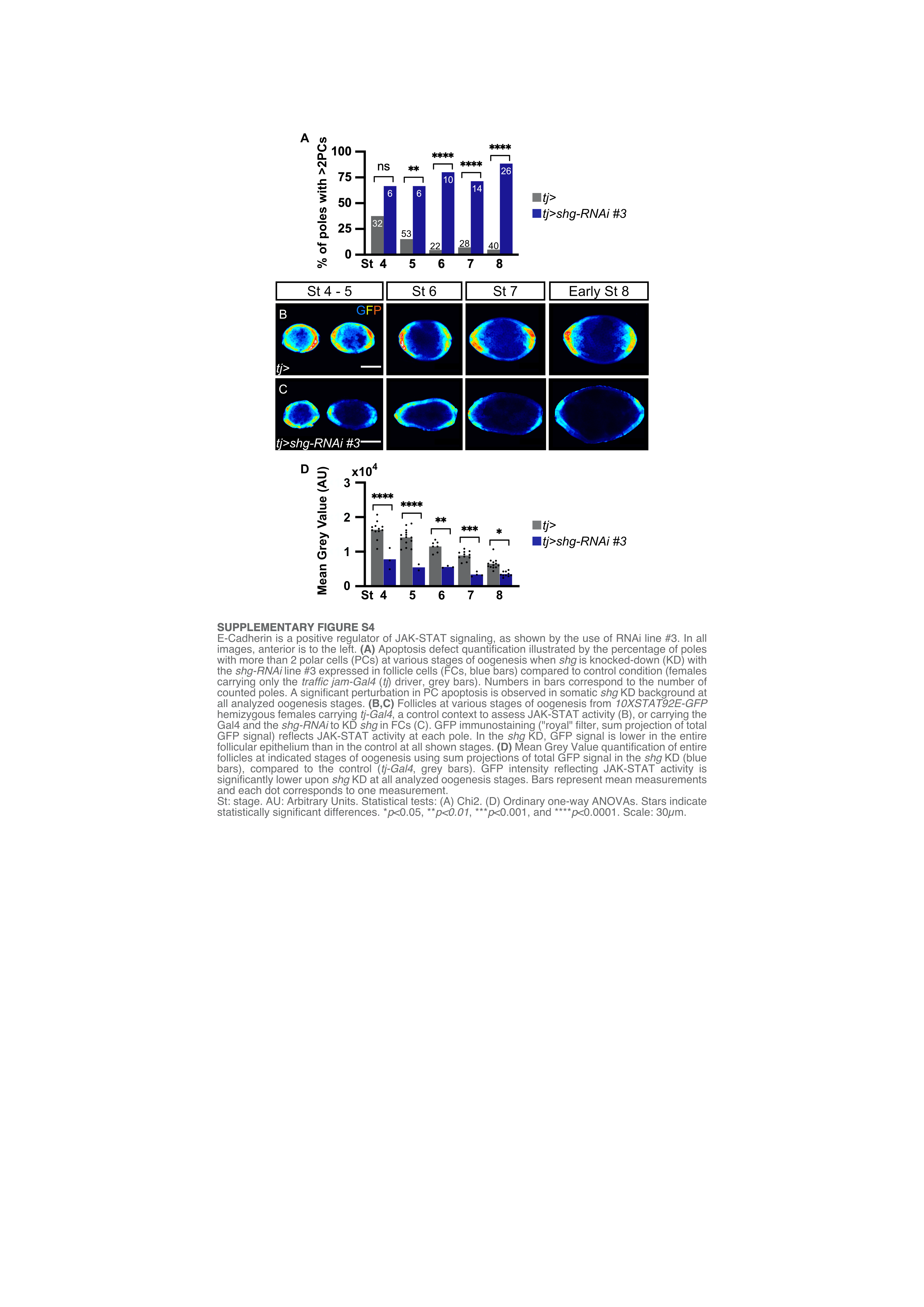

Supplement: Supplementary file 4 [file Image4.tiff]
